# Fingerprints of a size-dependent crossover in the dimensionality of electronic conduction in Au-seeded Ge nanowires

**DOI:** 10.3762/bjnano.7.151

**Published:** 2016-11-02

**Authors:** Maria Koleśnik-Gray, Gillian Collins, Justin D Holmes, Vojislav Krstić

**Affiliations:** 1 Department of Physics, Friedrich-Alexander-University Erlangen-Nürnberg (FAU), Staudtstr. 7, 91058 Erlangen, Germany; 2 Materials Chemistry & Analysis Group, Department of Chemistry, University College Cork, Cork, Republic of Ireland

**Keywords:** electrical transport, germanium nanowires, quasi-1D confinement, screening length, VLS growth

## Abstract

We studied the electrical transport properties of Au-seeded germanium nanowires with radii ranging from 11 to 80 nm at ambient conditions. We found a non-trivial dependence of the electrical conductivity, mobility and carrier density on the radius size. In particular, two regimes were identified for large (lightly doped) and small (stronger doped) nanowires in which the charge-carrier drift is dominated by electron-phonon and ionized-impurity scattering, respectively. This goes in hand with the finding that the electrostatic properties for radii below ca. 37 nm have quasi one-dimensional character as reflected by the extracted screening lengths.

## Results and Discussion

Synthetic germanium nanowires (Ge NWs) have been proposed as potential next-generation components for high-performance applications [1–3]. Besides representing prospective field-effect devices [4–5], they have attracted interest as building blocks for nanoscaled electrooptical components [6] and, due to their specific surface properties [4] can be envisaged as high-potential chemical and biological sensors as discussed for other types of semiconductor NWs [7–10].

Successful implementation of NWs into the aforementioned sensor technologies requires an optimized operation regime which will depend on the dimensionality of the electronic system. In particular, to ensure a significant electronic response to changes at the NW surface region, both the electrostatic screening length should be larger than the NW radius and the surface-to-volume ratio maximised [8]. This in turn will only be fulfilled for a certain range of screening lengths [7] and associated NW surface-to-volume ratios [8]. While the surface-to-volume ratio scales with NW radius and therefore can be controlled by adjusting synthesis conditions [11–12], the screening length depends on the density and dimensional character of the charge carriers in the NW [8]. Therefore it is crucial to investigate the charge transport properties in the NWs of choice as function of their radius *R* to identify their different operation regimes.

To this end, we carried out electrical characterization at ambient conditions of individual Au-seeded Ge NWs with *R* ranging from 11 to 80 nm (cf. Supporting Information File 1). By experimentally measuring the electrical conductivity, σ_NW_, and field effect mobility, μ_NW_, we were able to identify the dominant scattering mechanisms and the *R*-dependence of the electrostatic screening length.

**Figure 1:**
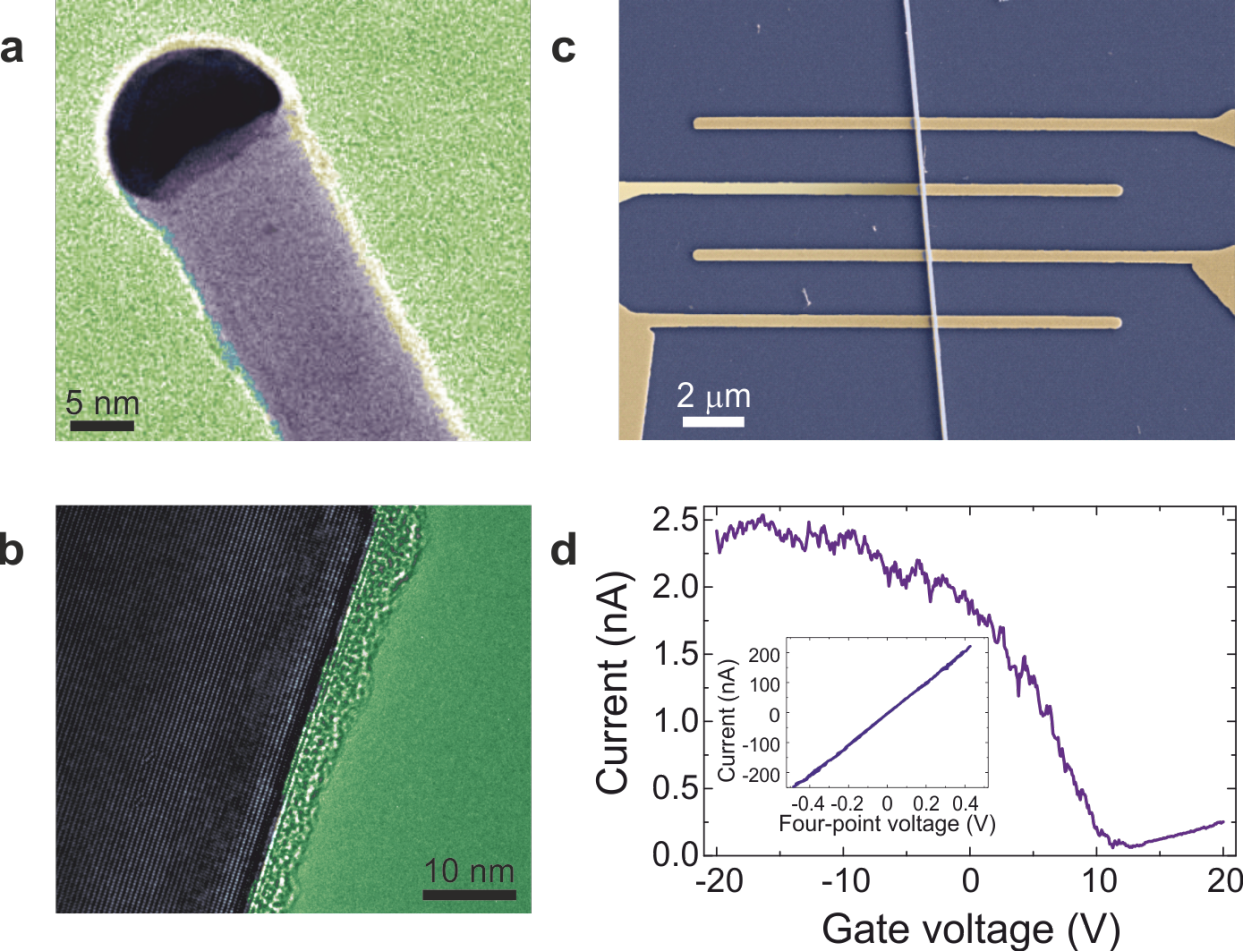
Characterization of Au-seeded Ge NWs. (a) TEM image of a Ge NW with an Au nanoparticle at the tip. (b) Higher magnification TEM image showing a NW with uniform crystalline structure covered by a thin native oxide layer. (c) SEM image of a four-terminal Ge NW device fabricated with 200 nm wide electrodes on top of a 30 nm radius wire. (d) Transfer curve taken at 0.5 V source-drain voltage for a 28 nm radius wire revealing *p*-type charge transport. Inset: four-point current–voltage characteristic of the same NW.

Ge NWs used in this study were synthesized at 400 °C on anodized alumina supports using a Au nanoparticle seeded vapour-liquid-solid process [13]. TEM analysis revealed that the NWs are monocrystalline with uniform radius along the axis (Figure 1a), have predominant <110> growth direction and are covered with a thin native oxide layer (Figure 1b). Individual NWs were deposited on degenerately doped Si substrates with 300 nm thermally grown SiO_2_ on top and contacted lithographically with Ag electrodes [14] in a four-point-probe configuration (Figure 1c). The Si backside of the chip was used as global backgate. For each NW, four-point current–voltage and transfer characteristics were taken under ambient conditions. All NWs showed *p*-type transfer characteristics (Figure 1d) indicating that the majority charge carriers are holes (cf. Supporting Information File 1) which is consistent with the existing studies on similar VLS grown semiconducting NWs [15–17]. In particular, the doping in our NWs is predominantly through surface states (cf. Supporting Information File 1). Also, our previous work [14] showed that the carrier-distribution is uniform over length scales of several hundred nm’s along each NW unlike potentially expected for deliberately volume-doped Si NWs [18–21].

**Figure 2:**
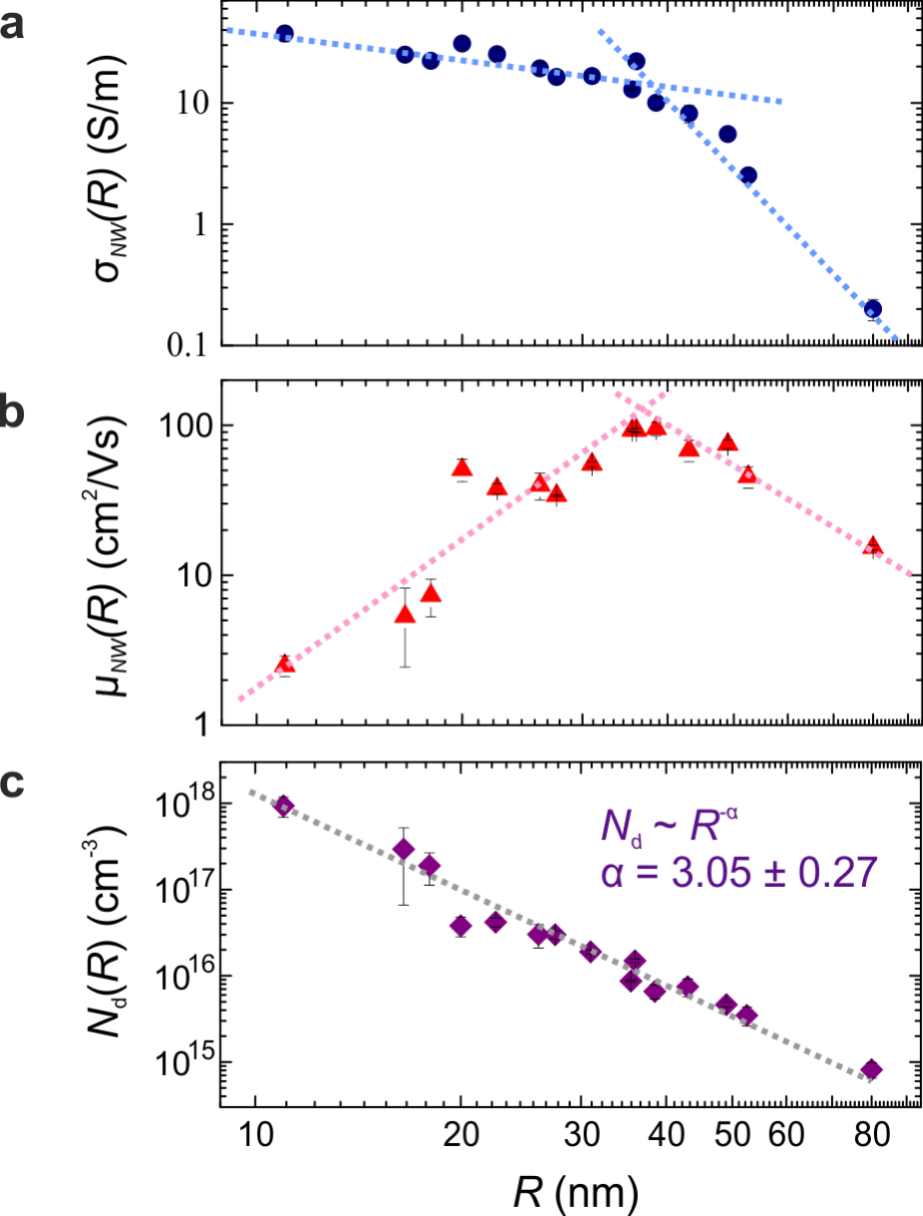
Radius-dependent charge transport properties in Ge NWs. (a) Electrical conductivity, (b) mobility and (c) charge carrier density as function of the NW radius *R*. Dotted lines in (a) and (b) are a guide to the eye. Grey dashed line in (c) corresponds to the numerical fitting of *N*_d_(*R*) with a power function.

From the measured data, σ_NW_, μ_NW_ and the carrier density, *N*_d_, values were extracted and are plotted in Figure 2 as function of the NW radius *R*.

σ_NW_(*R*) showed a monotonous decrease by two orders of magnitude with increasing NW radius (Figure 2a). A change in the *R*-dependence was observed at about 36 to 37 nm. Notably, for the same *R* we find a maximum in the μ_NW_(*R*) (Figure 2b). This qualitative change in the *R*-dependence for both σ_NW_(*R*) and μ_NW_(*R*) suggests a crossover in charge-carrier conduction. We note that the data in Figure 2a also confirms that surface-scattering is negligible at these radii, as the conductivity increases by orders of magnitude with radius reduction (an opposite dependence would have been expected otherwise).

To provide insight into the nature of this crossover, we first extract *N*_d_(*R*) (cf. Supporting Information File 1) which is shown in Figure 2c. The measured carrier density in all cases exceeds the intrinsic doping level of bulk Ge (1.3 × 10^13^ cm^−3^ [22]), which indicates that the carrier-concentration is equivalent to the number density of ionized acceptor levels (surface–dopant concentration). Numerical fitting revealed *N*_d_(*R*) ~ *R*^−α^ with α = 3.05 ± 0.37 showing that large radius NWs are comparably lightly doped. We note that the carrier concentration in the NW depends on the surface-state density which can vary depending on the synthesis conditions [15,23–24]. Therefore, the radius dependence of the carrier concentration may vary in differently grown NWs.

**Figure 3:**
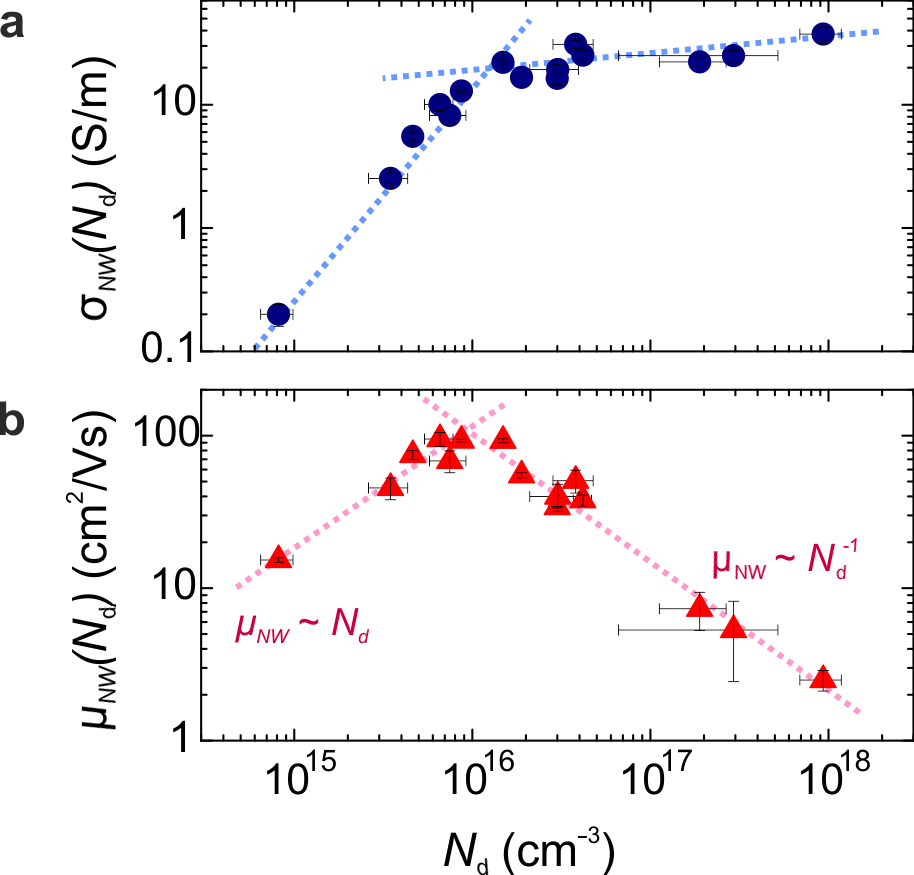
Carrier-density dependent transport properties in Ge NWs. (a) Electrical conductivity and (b) mobility as function of carrier density *N*_d_. Dashed lines are a guide to the eye.

Since we found a non-trivial *N*_d_(*R*) dependence, it is instructive to graph the conductivity and mobility as a function of carrier density, as is shown in Figure 3.

For low dopant densities, σ_NW_(*N*_d_) first increases by two orders of magnitude, and for *N*_d_ exceeding ≈10^16^ cm^−3^ it enters into a slowly varying regime (Figure 3a). In the case of mobility (Figure 3b), between ≤10^15^ and ≈10^16^ cm^−3^ μ_NW_(*N*_d_) ≈ *N*_d_, indicating that lattice phonon scattering is the main mechanism limiting the carrier drift [25]. The dominance of electron phonon scattering within this density range suggests that the free holes behave similar to those in (*p*-type) bulk Ge [18]. For higher *N*_d_, however, 
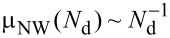
 which is characteristic of ionized impurities being the dominant scatterers [25]. This coincides with the slow-varying region in σ_NW_(*N*_d_) which indicates that a further augmentation in σ_NW_(*N*_d_) is inhibited due to the increased density of scattering centres which counterbalances the increasing *N*_d_. Remarkably, in bulk *p*-Ge ionized impurity scattering is expected to contribute significantly over our entire experimental carrier-density range [22,25–26], which would, in contrast to our data, lead to a rather flat *N*_d_ dependence up to 10^16^ cm^−3^.

Having found the dominant scattering contributions for the limit of low and high-carrier densities in the NWs, we now address the electrostatic screening length. Assuming first that the charge carriers in the NWs follow a 3D-type of behaviour over the entire *N*_d_ range, we determine the corresponding 3D Debye (screening) length, 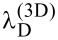, which is defined as [27]:

[1]
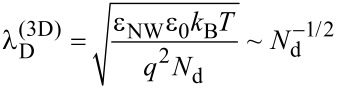


where ε_NW_ = 16 is the dielectric constant of the NW material (assumed the same as for bulk Ge [28]), ε_0_ the vacuum permittivity, *k*_B_ the Boltzmann constant, *T* the temperature and *q* the electron charge.

**Figure 4:**
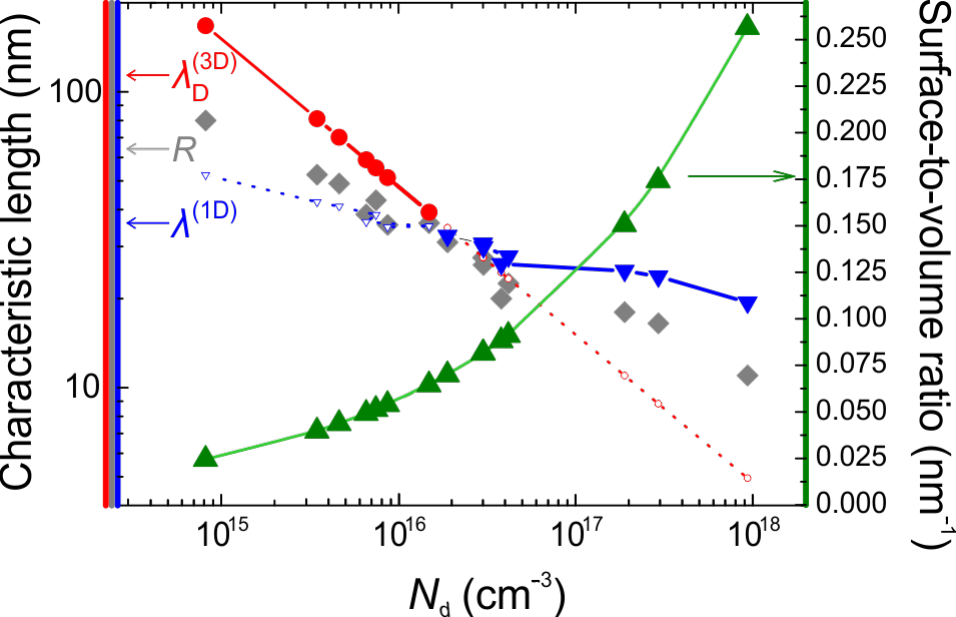
Characteristic length scales in Ge NWs. 3D Debye 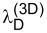 and 1D screening length λ^(1D)^ as function of carrier density *N*_d._ NW radius sizes (grey symbols) are plotted as a reference. Green triangles show the corresponding surface-to-volume ratios.

In Figure 4, 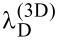 is plotted together with *R* as a function of *N*_d_. For low-carrier densities, 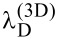 is larger than the NW radius and shows a continuous decrease until it becomes comparable to *R* at about 1 to 2 × 10^16^ cm^−3^. From this point on 
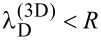
. However, a continued decrease in screening length would mean a reduction in the scattering cross-section, which is contradictory to the experimentally observed decrease in mobility (Figure 3b). Therefore this indicates that for higher *N*_d_ (smaller *R*) a 3D description of the electrostatics in the NW is not suitable anymore and thus the screening length has to be described by a lower dimensional scenario. Since in a NW a 2D description of the charge carriers is not a reasonable approach, the 1D screening length λ^(1D)^ is better suited [29]. For our NWs, we can write

[2]
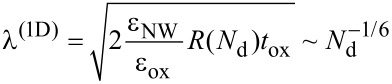


where ε_ox_ ≈ 7.44 [27] is the dielectric constant of the native oxide, *t*_ox_ ≈ 3 nm (cf. Figure 1b) its thickness, and *R*(*N*_d_) is the inverted *N*_d_(*R*) ~ *R*^−α^ (see above). That is, the screening length changes much slower with *N*_d_ compared to the 3D case.

Plotting λ^(1D)^ also in Figure 4 we find a seamless matching of λ^(1D)^ and 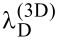 at about 1 to 2 × 10^16^ cm^−3^ (equivalently *R* ≈ 35 to 38 nm) which falls close to both the maximum in μ_NW_(*N*_d_) and the entering into the slow varying regime of σ_NW_(*N*_d_) (Figure 3). We note that with increasing *N*_d_, λ^(1D)^ stays well above *R* which is consistent with the mobility decrease as well as the observed ionized impurity scattering dominating in this regime.

Considering the potential application of the Au-seeded Ge NWs for sensors, Figure 4 also reveals the most suitable range of radii when plotting the surface-to-volume ratio also. Clearly, the quasi-1D regime is preferred as there the screening length is larger than *R* and the surface-to-volume ratio is maximised. In contrast, for radii ≥37 nm, the surface-to-volume ratio is by orders of magnitude lower, that is, not both prerequisites for an optimum sensor operation are met.

Summarizing, we demonstrated that the dominant scattering mechanisms and the electrostatic screening properties of Au-seeded VLS grown Ge NWs at room temperature are strongly dependent on their radius. Our results show that a crossover in charge carrier conduction occurs for carrier densities exceeding ≈10^16^ cm^−3^, equivalent to the radius decreasing below approximately 37 nm. Analysis of the electrical screening properties shows that this is associated with a shift from a 3D to quasi-1D regime where the carrier drift is limited predominantly by ionized impurity scatterers. This suggests that Ge NWs only in the quasi-1D regime can be expected to deliver high-performance sensor capabilities.

## Supporting Information

File 1 Extraction of intrinsic electrical transport parameters from measurement.
