# Supplementary material for: Fingerprints of a size-dependent crossover in the dimensionality of electronic conduction in Au-seeded Ge nanowires
Source: Beilstein J Nanotechnol. 2016 Nov 2;7:1574–8. doi: 10.3762/bjnano.7.151 (PMC5238664; doi:10.3762/bjnano.7.151)
Supplement: File 1 — Extraction of intrinsic electrical transport parameters from measurement. [file Beilstein_J_Nanotechnol-07-1574-s001.pdf]

Supporting Information

for

Fingerprints of a size-dependent crossover in the dimensionality of electronic conduction in Au-seeded Ge nanowires

Maria Koleśnik-Gray[1], Gillian Collins[2], Justin D. Holmes[2] and Vojislav Krstić[*1]

Address: [1]Department of Physics, Friedrich-Alexander-University Erlangen-Nürnberg (FAU), Staudtstr. 7, 91058 Erlangen, Germany and [2]Materials Chemistry & Analysis Group, Department of Chemistry, University College Cork, Cork, Republic of Ireland

Email: Vojislav Krstić - vojislav.krstic@fau.de

* Corresponding author

Extraction of intrinsic electrical transport parameters from measurement

A. Nanowire conductivity

Nanowire resistance is measured in a four-point probe configuration, and is given by $R_{NW} = V_{4T} / I_d$ where V_{4T} is the voltage drop measured between the inner electrodes and I_d the current. For a NW of radius *R*, the *conductivity* is given by

$$\sigma_{NW} = \frac{1}{R_{NW}} \frac{L_C}{\pi R^2} \quad , \tag{S1}$$

where L_C is the conduction channel length, defined as the distance between the outer edges of the four-point probe electrodes (cf. Ref [1] for more details).

B. Carrier mobility

Charge carrier mobility of the NWs was measured via the field-effect where the number of charges in the conduction channel is externally controlled by the applied gate voltage V_G. The electrical response of a NW device is quantified by its *extrinsic transconductance* g_m which is found from the sub-threshold slope of a transfer curve at fixed source-drain voltage V_{SD}:

$$g_m \equiv \left| \frac{\partial I_d}{\partial V_G} \right|_{V_{sd}} . \qquad \text{(S2),}$$

The *intrinsic NW transconductance* can be obtained by decoupling the device-specific contact resistances R_C:

$$g_{in} = g_m \frac{1 + \frac{R_C}{R_{NW}}}{1 - g_m R_C / 2}, \qquad \text{(S3)}$$

assuming that both source- and drain-electrode contact resistances are equivalent.
The contact resistance in turn is measured in two point configuration, where the total device resistance is given by: $R_{Tot} = R_{NW} + R_C$.

The *intrinsic mobility* can thus be found using the expression:

$$\mu_{NW} = \frac{g_{in}}{V_{SD}} \frac{L_C^{\ 2}}{C}, \qquad \text{(S4),}$$

where the capacitance *C* of the NW to the gate electrode is described by:

$$C = 2\pi \varepsilon_{SiO_2} \varepsilon_0 \frac{L_C}{\ln(t_{ox} / R)} \qquad \text{(S5)}$$

with t_{ox} = 300 nm the SiO_2 thickness and ε_{SiO_2} its dielectric constant.

C. Carrier density

Carrier density can be calculated as:

$$N_d = \frac{\sigma_{NW}}{q \mu_{NW}} \qquad \text{(S6)}$$

with *q* the electron charge.

Dominant doping from surface-states

Proof for the circumstance that we have doping which is dominated by the surface-states of the nanowire is seen by the following consideration:

We work with Au-seeded VLS Ge nanowires. From the entire synthesis process [2], one could assume that Au atom dopants – which would then be volume-dopants – provide the charge-carriers which we have measured in our experiments.

However, Au dopants in Ge are deep-level acceptors with an activation energy of the order of 100 to 200 meV (e.g. [3-5]). That is, their ionisation probability at room temperature (our experimental condition) would be below 1%. Considering our experimentally measured carrier densities between 10^{15} to 10^{18} cm^{-3}, we would have 10^{17} to 10^{20} cm^{-3} Au-atom density in the Ge nanowires. This large amount of Au atoms however has not been detected in TEM of our nanowires (cf. Figure 1b; [2]) but would have been easily detectable by TEM techniques (see e.g. [6,7])

Weak switching behaviour of Ge NWs (transfer characteristics)

The poor switching behaviour is typical for VLS synthesized Ge NWs and is a consequence of the generally high doping of the NWs through surface states up to 10^{18} cm^{-3} (several orders of magnitude higher than intrinsic Ge).

Supplementary References:

1. Koleśnik-Gray, M.; Lutz, T.; Collins, G.; Biswas, S.; Holmes, J.D.; Krstić, V. *Appl. Phys. Lett.* **2013,** *103*, 153101.
2. Collins, G.; Koleśnik, M.; Krstić, V.; Holmes, J.D. *Chem. Mater.* **2010,** *22*, 5235-5243.
3. Woodbury, H.H. & Tyler, W.W. Phys. Rev. 1957, 105, 84-92.4. Sze, S.M. *Physics of Semiconductor Devices*, 2nd ed.; Wiley 1981.
4. Dunlap, W.C. Phys. Rev. 1955, 97, 614-629.
5. Chapter 5 in: Egerton, Electron Energy-Loss in the Electron Microscope, Ch. 5
6. Allen, J.E. et al. Nature Nanotech. **2008,** *8,* 168-173.
